# YOLOv8-LCNET: An Improved YOLOv8 Automatic Crater Detection Algorithm and Application in the Chang’e-6 Landing Area

**DOI:** 10.3390/s25010243

**Published:** 2025-01-03

**Authors:** Jing Nan, Yexin Wang, Kaichang Di, Bin Xie, Chenxu Zhao, Biao Wang, Shujuan Sun, Xiangjin Deng, Hong Zhang, Ruiqing Sheng

**Affiliations:** 1 State Key Laboratory of Remote Sensing Science, Aerospace Information Research Institute, Chinese Academy of Sciences, Beijing 100101, China; nanjing23@mails.ucas.ac.cn (J.N.); dikc@aircas.ac.cn (K.D.); xiebin21@mails.ucas.ac.cn (B.X.); zhaochenxu22@mails.ucas.ac.cn (C.Z.); wangbiao221@mails.ucas.ac.cn (B.W.); 2 University of Chinese Academy of Sciences, Beijing 100101, China; 3 Center for Excellence in Comparative Planetology, Chinese Academy of Sciences, Hefei 230026, China; 4 School of Architecture and Civil Engineering, Chengdu University, Chengdu 610106, China; sunshujuan@cdu.edu.cn; 5 Beijing Institute of Spacecraft System Engineering, China Academy of Space Technology, Beijing 100094, China; dengxiangjin@sina.com (X.D.); zhanghong122078@163.com (H.Z.); shengruiqing1984@163.com (R.S.)

**Keywords:** lunar surface, CE-6 landing area, digital orthophoto map, impact crater, automatic detection, You Only Look Once-v8

## Abstract

The Chang’e-6 (CE-6) landing area on the far side of the Moon is located in the southern part of the Apollo basin within the South Pole–Aitken (SPA) basin. The statistical analysis of impact craters in this region is crucial for ensuring a safe landing and supporting geological research. Aiming at existing impact crater identification problems such as complex background, low identification accuracy, and high computational costs, an efficient impact crater automatic detection model named YOLOv8-LCNET (YOLOv8-Lunar Crater Net) based on the YOLOv8 network is proposed. The model first incorporated a Partial Self-Attention (PSA) mechanism at the end of the Backbone, allowing the model to enhance global perception and reduce missed detections with a low computational cost. Then, a Gather-and-Distribute mechanism (GD) was integrated into the Neck, enabling the model to fully fuse multi-level feature information and capture global information, enhancing the model’s ability to detect impact craters of various sizes. The experimental results showed that the YOLOv8-LCNET model performs well in the impact crater detection task, achieving 87.7% Precision, 84.3% Recall, and 92% AP, which were 24.7%, 32.7%, and 37.3% higher than the original YOLOv8 model. The improved YOLOv8 model was then used for automatic crater detection in the CE-6 landing area (246 km × 135 km, with a DOM resolution of 3 m/pixel), resulting in a total of 770,671 craters, ranging from 13 m to 19,882 m in diameter. The analysis of this impact crater catalogue has provided critical support for landing site selection and characterization of the CE-6 mission and lays the foundation for future lunar geological studies.

## 1. Introduction

**Figure 1:**
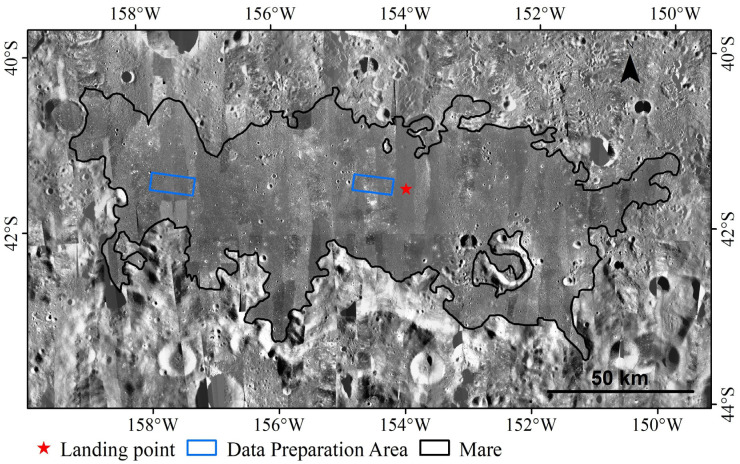
CE-6 landing area DOM with a resolution of 3 m/pixel.

On 25 June, the Chang’e-6 (CE-6) return capsule accurately landed in the predetermined area of the Siziwang Banner, Inner Mongolia, marking the complete success of the CE-6 mission, and achieving the world’s first lunar farside sample return mission [1]. The landing point of CE-6, with a precise position of (153.9856° W, 41.6383° S) in the LROC NAC digital orthophoto map (DOM) [2], as shown in Figure 1, is located in the southwestern part of the Apollo Basin within a lunar mare, near the northeastern edge of the SPA Basin. This location is potentially close to an area where lunar mantle material is exposed, offering the collected samples rich geological information [3,4]. These samples from the far side of the Moon are of great significance for addressing key scientific issues related to the Moon’s internal composition and structure, impact history, and the development of lunar habitats, etc. [5,6,7,8,9,10].

The surface of the Apollo basin is covered with impact craters of various morphologies. Studying these impact craters, including the size–frequency distribution and spatial density analysis, can assist the CE-6 lander navigate and avoid obstacles for a safe landing in complex terrain [11,12,13]. Additionally, these analyses, combined with radiometric ages from collected samples, can refine the lunar chronology model and provide a more accurate lunar chronology model [14,15,16]. These studies will contribute to understanding the impact processes of the Apollo basin and the SPA basin, and the geologic evolution of the Moon.

Lunar impact craters exhibit complex and diverse morphologies, with a wide range of scales. When the resolution of remote sensing images improves from the 100-m scale to the meter scale, the number of impact craters will show an order-of-magnitude increase. Although the manual extraction of craters can achieve high accuracy, it is time-consuming and labor-intensive, making it difficult to meet the demands of large-scale data processing. This presents a great challenge for extracting a huge number of small and medium-sized craters from high-resolution remote sensing images. Therefore, the automatic extraction of impact craters has become an important research direction in the field of planetary remote sensing.

Automatic impact crater detection methods can be broadly classified into traditional methods and deep-learning-based methods. Traditional crater detection methods mainly rely on digital image processing techniques, including edge detection methods [17,18,19], morphology-based feature extraction methods [20,21,22] and traditional machine learning methods [23,24,25], etc. These methods extract the ring-like features of craters from orbiter images or digital elevation models, but are only effective for craters with relatively clear boundaries and are easily affected by noise, shadows, and other factors, leading to limited recognition accuracy.

Deep learning has rapidly advanced in recent years and demonstrates significant advantages in the field of object detection [26,27,28]. Currently, deep-learning-based detection frameworks are divided into two-stage detection frameworks and one-stage detection frameworks. Two-stage detection algorithms are represented by R-CNN [29], Faster R-CNN [30], Sparse R-CNN [31], etc., which need to generate candidate regions first, then perform object category prediction and more accurate bounding box regression on the regions. In contrast, one-stage detection algorithms directly predict the category and location of the target without pre-generating the candidate region [32,33]. Compared to two-stage algorithms, one-stage object detection algorithms are simpler and faster. As a result, numerous networks using one-stage detection algorithms for the recognition of planetary surface impact craters have emerged, such as CraterNet [34]; AE-TransUNet+ [35]; DeepMoon [36]; YOLO-Crater [37]; and HRFPNet [38].

The above studies have made progress in automatic impact crater detection, but there are still many difficulties and challenges in complex scenarios, especially when faced with the multi-scale characteristics of impact craters and the dense distribution of small craters, which can easily result in false and missed detections. To address these issues, this paper proposes an efficient impact crater detection model, YOLOv8-LCNET (YOLOv8-Lunar Crater Net), based on the one-stage object detection algorithm YOLOv8 (https://github.com/ultralytics/ultralytics, accessed on 15 June 2023). YOLOv8n was selected due to its balance between detection speed and accuracy, making it suited for the real-time processing demands of crater detection. Hence, all subsequent mentions of YOLOv8 in this paper refer to YOLOv8n. The main contributions of this work are as follows:

(1) An impact crater dataset consisting of 84,480 images, with each at 512 × 512 pixels in size, was created. The data was obtained by cropping from two high-resolution local DOMs (15 km × 6 km each) with better than 1 m/pixel resolution within the CE-6 landing area. And, data augmentation techniques such as flipping, affine transformation, and image enhancement were applied to increase sample diversity;
(2) The YOLOv8 algorithm was improved by incorporating the Partial Self-Attention (PSA) module [39] and Gather-and-Distribute mechanism (GD) [40], and ablation experiments were conducted to demonstrate the effectiveness of these additions. Experimental results show that the improved YOLOv8 model achieved an accuracy of 87.7%, a recall rate of 84.3%, and an average accuracy of 92% in the impact crater detection task. Compared with the original YOLOv8 model, these metrics increased by 24.7%, 32.7%, and 37.3%, respectively;
(3) A database of impact craters in the CE-6 landing area (246 km × 135 km) extracted by YOLOv8-LCNET was established based on the DOM mosaic with a resolution of 3 m/pixel, containing a total of 770,671 impact craters, of which 511,520 craters were larger than the completeness diameter of 30 m. Analysis of this database provided support for both engineering applications and scientific research.

## 2. Methods

### 2.1. YOLOv8-LCNET

**Figure 2:**
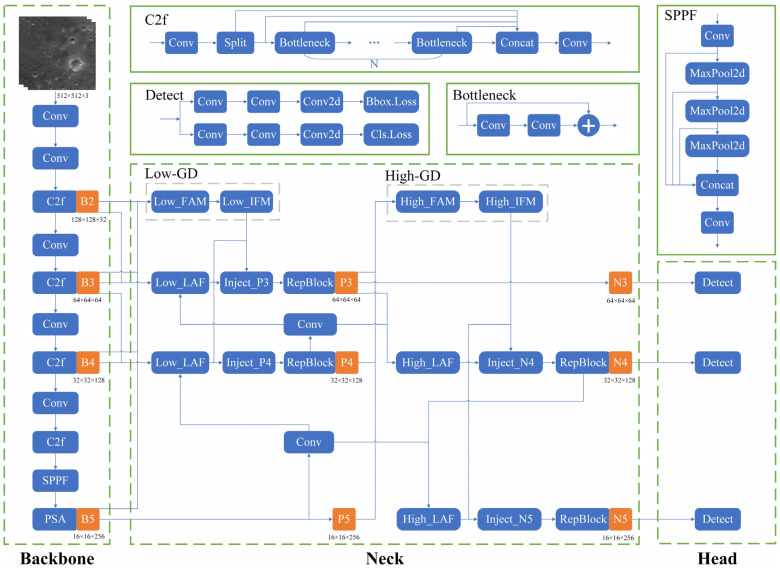
The structure of YOLOv8-LCNET. {B2, B3, B4, B5}, {P3, P4, P5}, and {N3, N4, N5} denote feature maps.

In this paper, we propose an improved lunar surface impact crater detection network YOLOv8-LCNET, based on the YOLOv8 algorithm. The overall architecture is shown in Figure 2.

The YOLOv8-LCNET consists of three main components: the Backbone, Neck, and Head. The Backbone is primarily responsible for impact crater feature extraction, the Neck handles feature fusion, and the Head is used to predict the location and size of the impact craters. The PSA module was adopted at the end of the Backbone part, which optimizes computational resources and reduces complexity while maintaining global modeling capability [39], thereby reducing the omission of impact craters. The GD mechanism of Gold-YOLO [40] was integrated in the Neck part, allowing the model to gather global information by fusing features from different layers and then injecting this information back into the features at various levels. This facilitated information interaction and fusion, improving the model’s ability to detect impact craters of varying sizes. Specific details on these components are discussed in subsequent sections.

### 2.2. PSA

After the final stage Spatial Pyramid Pooling Fast (SPPF) module of the Backbone, we added an efficient PSA attention mechanism [39]. The PSA module is an improvement over the traditional self-attention mechanism, designed to reduce computational complexity and memory usage while retaining global modeling capability. This mechanism divides the input feature map (16 × 16 × 256) into two equal parts: one part (16 × 16 × 128) undergoes self-attention processing, while the other (16 × 16 × 128) bypasses it.

Specifically, the portion subjected to self-attention was processed using Multi-Head Self-Attention (MHSA) and Feed-Forward Network (FFN) [41]. Additionally, the dimensions of the Key and Query inputs in the MHSA module were reduced to half of the Value dimension, and Batch Normalization (BatchNorm) [42] was used to replace Layer Normalization (LayerNorm) [43] to speed up inference. These optimizations lowered the computational complexity associated with self-attention, ensuring efficient processing while maintaining the mechanism’s ability to model long-range dependencies.

By applying this partial self-attention computation, the PSA mechanism achieved a balance between performance and efficiency, making it suited for computationally intensive tasks, such as the detection of lunar impact craters.

### 2.3. GD Mechanism

In the Neck section, the GD mechanism was adopted to enhance the model’s ability to integrate and utilize global information [40]. By integrating features across multiple levels and redistributing this global information back to various feature layers, the GD mechanism facilitates information interaction and fusion. This approach improves the model’s capacity to detect impact craters of varying sizes without notably increasing latency.

As shown in Figure 2, the GD mechanism was divided into two branches: the low-stage gather-and-distribute branch (Low-GD) and the high-stage gather-and-distribute branch (High-GD), designed to process shallow and deep features, respectively, ensuring that both low-level and high-level features were utilized. Both branches followed the implementation process: starting with the Feature Alignment Module (FAM), which gathers and aligns features from different layers. Specifically, the FAM module gathers shallow features from the Backbone layers {B2, B3, B4, B5} or deep features from feature maps {P3, P4, P5} processed by the Low-GD branch. Next, the Information Fusion Module (IFM) fuses the gathered features using convolutional operations in Low-GD and transformer-based operations in High-GD to obtain global information. Finally, the Information Injection Module (Inject) distributes this global information back into each level by delivering the fused feature information to {P3, P4} in Low-GD and {N4, N5} in High-GD, ensuring the integration of both local and global features across different scales. This hierarchical process enhanced the model’s ability to detect objects of various sizes while balancing accuracy and computational efficiency.

## 3. Experiments

### 3.1. Dataset

**Figure 3:**
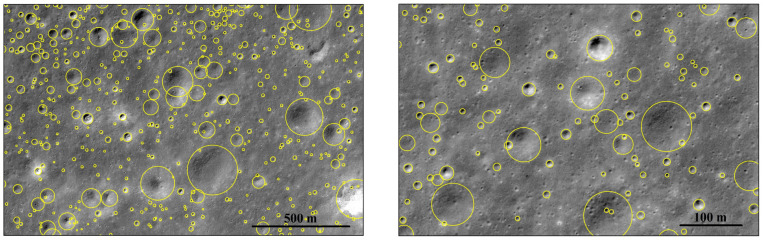
Some sample results of impact crater extraction from two local DOM mosaics.

In this study, two local DOM mosaics, each covering an area of 15 km × 6 km within CE-6 landing area, as shown in Figure 1, with resolutions of 0.86 m/pixel and 0.6 m/pixel, respectively [15], were selected to establish an impact crater sample dataset. A total of 66,030 craters with diameters ranging from 3.7 m to 609 m were extracted from the two DOM mosaics. Some examples of the extraction results are shown in Figure 3. These mosaic DOM images were then cropped into three-channel sub images of 512 × 512 pixels with the greyscale values stretched to [0, 255] to help the network converge. Meanwhile, the sub images are required to have a certain overlapping region during cropping to reduce the likelihood of impact craters being cut off at the cropping boundary, thereby improving the completeness of the contours [44].

In addition, in order to improve the generalization capability of YOLOv8-LCNET, random data augmentation techniques such as flipping, affine transformation, and histogram enhancement were applied. Specifically, horizontal flipping was applied with a probability of 50%, and vertical flipping with a probability of 20%. Affine transformations included scaling in the range of 80% to 120% and rotation within ±45 degrees. Additionally, pixel intensity adjustments were adopted, including sharpening (with an alpha range of 0 to 1.0 and a lightness range of 0.75 to 1.5), multiplicative changes in pixel intensity ranging from 0.5 to 1.5, and Contrast Limited Adaptive Histogram Equalization [45] to enhance local contrast. These augmentation techniques resulted in the creation of a dataset comprising 84,480 sub-images and their corresponding labels. This dataset was then divided into training and validation sets at the ratio of 8:2 for model training and performance evaluation.

### 3.2. Experimental Platform and Parameters

The hardware environment for the experiment included an NVIDIA GeForce RTX 3090 GPU and 96 GB of RAM. The deployment environment for the experiment involved CUDA 12.2, Python 3.9, and PyTorch 2.3.1 + cu121.

The hyperparameters for the experiment were set as follows: the learning rate was set to 0.01, the momentum was 0.937, the weight decay coefficient was 0.0005, the batch size was 64, and the number of iterations was 300. To ensure the comparability of the experimental results, all experiments in this study were conducted in the same environment and with identical parameter settings.

### 3.3. Evaluation Metrics

In order to evaluate the accuracy of the algorithmic models, performance metrics for object detection algorithms [46] were used in this paper. Both the Intersection over Union (IoU) and confidence parameters were set to 0.5, and the evaluation metrics included Precision (P), Recall (R), and Average Precision (AP50, AP50−95, $AP_{S}$, $AP_{M}$, $AP_{L}$). As shown in Figure 3, not all impact craters were labelled. Due to the large number of craters, many remained unlabeled, which led to these unlabeled craters being considered as false positives. This situation made precision-based evaluation challenging. Therefore, to make the evaluation results more convincing, this study adopted the false positive rate of impact crater identification, also known as the Discovery Rate (DR), which was defined as the ratio of the number of newly discovered unlabeled craters to the number of all impact craters (recognized and unrecognized craters) [47].

(1) $$DR=\frac{FP}{FP+TP+FN}$$

where FP represents false positives, i.e., the number of impact craters predicted that do not match ground-truth craters; TP represents true positives, i.e., the number of impact craters correctly predicted in the ground-truth crater set; and FN represents false negatives, i.e., the number of impact craters not detected in the ground-truth crater set.

In addition to the primary evaluation metrics for the impact crater identification described above, the accuracy of the position and diameter size of the impact craters were also important metrics for assessing model performance. The fractional errors of the latitude and longitude as well as the radius of the impact craters were calculated using the following formula [36].

(2) $$\left\{\begin{array}{l} dLo/R=abs(Lo_{P}-Lo_{G})\cos(\pi \langle La\rangle /180{}^{\circ})/(R_{G}C_{KD})\\ dLa/R=abs(La_{P}-La_{G})/(R_{G}C_{KD})\\ dR/R=abs(R_{P}-R_{G})/R_{G} \end{array}\right.$$

where, $Lo_{P}$ and $Lo_{G}$ refer to the longitude of the center of the predicted and actual impact craters, respectively, while $La_{P}$ and $La_{G}$ refer to the latitude of the center of the predicted and actual impact craters, respectively. $\langle La\rangle =\left(La_{P}+La_{G}\right)/2$, $C_{KD}=180{}^{\circ}/(\pi R_{Moon})$ is the conversion factor from kilometers to degrees, where $R_{Moon}$ represents the radius of the Moon in km. $R_{P}$ and $R_{G}$ refer to the predicted and actual radius of the impact crater, respectively.

Additionally, Giga Floating-point Operations (GFLOPs) and Frames Per Second (FPS) were reported separately to evaluate the model’s efficiency.

### 3.4. Experimental Results

#### 3.4.1. Data Augmentation Experiments

Remote sensing images in natural environments are often affected by a variety of factors such as lighting changes, shadows, noise, etc., which may lead to a decrease in the recognition accuracy of the model in practical applications. To improve the model’s robustness and generalization ability, this study applied data augmentation methods described in Section 3.1. Table 1 shows the differences in YOLOv8 performance before and after applying these data augmentation techniques to assess the impact of data augmentation on model performance.

It is obvious from the experimental results that the overall performance of the YOLOv8 model on the validation set was significantly improved after applying data augmentation, with the model’s P improved by 0.228 and R improved by 0.312. The data augmentation enabled the model to learn a more diverse set of feature representations, enhancing its ability to handle diverse and unpredictable conditions. As a result, the model trained with augmented data maintained higher recognition accuracy when dealing with complex situations in real-world tasks.

#### 3.4.2. Ablation Experiments

To verify the effectiveness of the proposed improvement modules, ablation experiments were conducted to evaluate the impact of different modules on the model’s detection performance. The evaluation results are shown in Table 2. These results provided a detailed comparison of the model’s performance with and without specific modules, helping to determine the contribution of each module to the overall improvement in detection accuracy.

As seen in the table, the YOLOv8 model with the PSA attention mechanism showed an improvement in DR, increasing by 0.026 compared to the original model, along with slight improvements in other performance metrics. This result indicated that the YOLOv8 model with the PSA attention mechanism reduced the leakage detection on the basis of improved accuracy, enhanced the model’s ability to capture important features such as crater edges and textures, and improved the model’s recognition accuracy on complex backgrounds. The YOLOv8 model with the GD mechanism also exhibited performance improvements in crater identification tasks, with $AP_{S}$, $AP_{M}$ and $AP_{L}$ increasing by 0.086, 0.115, and 0.096, respectively. These three metrics correspond to the average precision for small craters (area <$32^{2}$ pixels), medium craters ($32^{2}$< area <$96^{2}$ pixels), and large craters (area >$96^{2}$ pixels) [46], respectively. This indicated that the improved model greatly enhanced the accuracy in detecting impact craters of different scales. Furthermore, adopting the PSA mechanism resulted in only a slight decrease in FPS and a slight increase in GFLOPs, which confirmed that the PSA mechanism had a minimal impact on computational load, allowing the model to remain efficient.

The incorporation of the PSA attention mechanism and the GD mechanism enhanced the performance of the YOLOv8 model at different levels. The PSA mechanism focused on improving the model’s sensitivity to key features and target detection capabilities while maintaining low computational cost. On the other hand, the GD mechanism enhanced the model’s accuracy and generalization performance in detecting different scales of craters. These improvements resulted in further enhancements of the YOLOv8-LCNET model across various evaluation metrics, enabling more accurate and comprehensive detection in crater identification tasks.

#### 3.4.3. Visualization of the Results and Analysis

**Figure 4:**
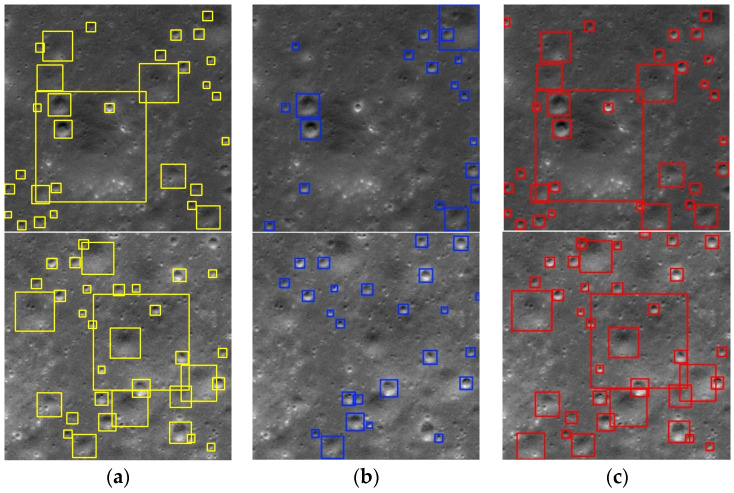
Comparison of the predicted impact crater detection results using YOLOv8 and YOLOv8-LCNET algorithms. (**a**) Ground-Truth, (**b**) YOLOv8, (**c**) YOLOv8-LCNET.

The original YOLOv8 algorithm as well as the YOLOv8-LCNET algorithm were used to detect the impact craters, and the predicted results are shown in Figure 4. The prediction results provided the relative size and centroid pixel coordinates of each predicted bounding box in each 512 × 512 pixel image. It was observed that compared to the original YOLO8, YOLOv8-LCNET is able to detect impact craters of various sizes and reduce missed detections.

**Figure 5:**
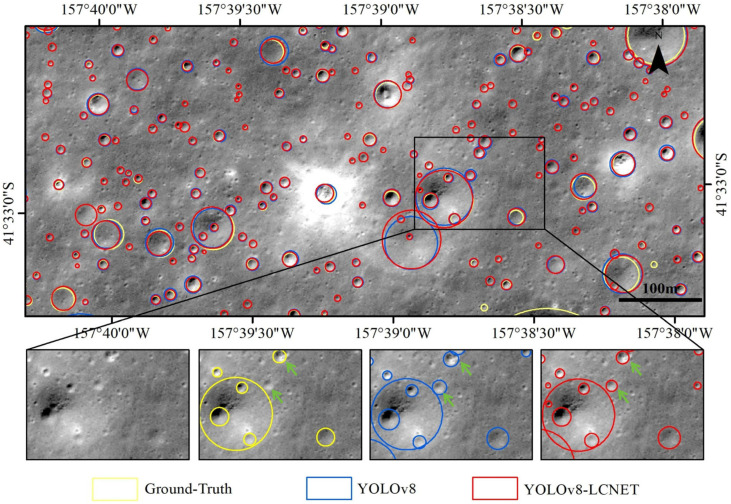
Comparison of the impact crater detection in a randomly selected area in SHP format.

To display the crater location information across the entire DOM image, the target detection results were converted into the ESRI Shapefile (SHP) format, with the center point coordinates converted to geographic coordinates. The crater diameter was defined as the diameter of a circle with an area equal to that of the ellipse formed by the predicted bounding box’s length and width. This diameter was then converted to the local coordinate system with the unit in kilometers, ensuring that the diameter remained consistent regardless of changes in the projection coordinate system. A randomly selected area is displayed in SHP format for comparison, as shown in Figure 5.

The yellow circles in the figures indicate the real labels of the impact craters, the blue circles are the impact craters predicted by the YOLOv8 algorithm, and the red circles are the impact craters predicted by the YOLOv8-LCNET algorithm. As seen in Figure 4 and Figure 5, the YOLOv8-LCNET algorithm nearly identified all labeled craters and detected a large number of newly discovered craters, in which the smallest ones were with diameters as small as four pixels. Furthermore, compared to the original YOLOv8 algorithm, the YOLOv8-LCNET algorithm predictions aligned more closely with the edges of the true boundaries.

In order to quantitatively compare the accuracy of the two algorithms in identifying the location and size of the impact craters, the fractional errors in latitude, longitude, and radius of the impact craters predicted by YOLOv8 and YOLOv8-LCNET were calculated, respectively. Additionally, to provide a comprehensive comparison of the differences before and after the improvements, performance metrics such as P, R, and other relevant evaluation metrics are also presented in Table 3.

As shown in Table 3, dLo/R, dLa/R, and dR/R were reduced by 11.2%, 10.0%, and 8.8%, respectively, calculated as ((YOLOv8’s metric—YOLOv8-LCNET’s metric)/YOLOv8’s metric) × 100%, demonstrating a substantial increase in the accuracy of crater extraction in terms of position and size with the improved YOLOv8 model.

#### 3.4.4. Network Performance Comparison

**Figure 6:**
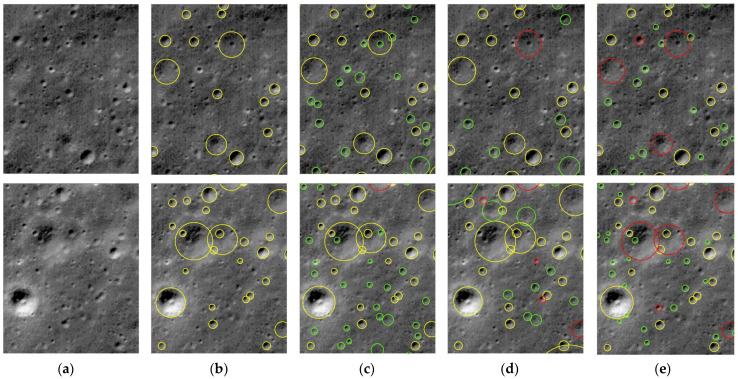
Comparison of the crater detection algorithms in different areas. The yellow circles stand for TP, the green circles stand for FP, which correspond to newly discovered unlabeled craters, and the red circles stand for FN. (**a**) Background, (**b**) Ground-Truth, (**c**) YOLOv8-LCNET, (**d**) Wang et al. [48], (**e**) Xie et al. [49].

Figure 6 illustrates the comparative results of the automatic crater detection using YOLOv8-LCNET and the algorithms proposed by Wang et al. [48] and Xie et al. [49].

Compared to the algorithms proposed by Wang et al. [48] and Xie et al. [49], YOLOv8-LCNET exhibited higher detection accuracy across craters of varying scales, making progress in addressing the challenges associated with multi-scale crater detection. In addition, YOLOv8-LCNET demonstrated greater robustness and stability in detecting less distinct or low-contrast craters, highlighting its capability to handle complex detection scenarios.

## 4. Impact Crater Catalogue in CE-6 Landing Area

The YOLOv8-LCNET algorithm was used to extract impact craters from the CE-6 landing area DOM mosaic with a resolution of 3 m/pixel, shown in Figure 1, resulting in a catalogue of craters with a Lambert conformal conic map projection. Subsequently, a local projection was applied to obtain the final impact crater positions and diameters, ensuring that the impact crater measurements were not affected by the map projection. Next, the ArcGIS extension tool “CraterTools” [50] was utilized to manually check and correct the detection results, fixing errors from the algorithm and adding missed craters. The final impact crater catalogue is provided in SHP format, with the attribute table recording the latitude and longitude coordinates of the impact crater centers, as well as their diameters, to support further scientific analysis and applications.

**Figure 7:**
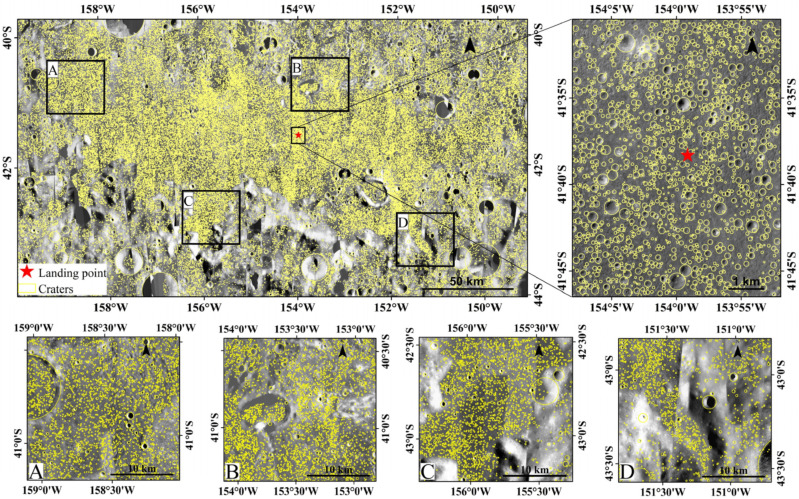
Impact craters with diameters greater than 120 m extracted from the CE-6 landing area, with subfigures showing the algorithm’s performance across four evenly distributed regions (**A**–**D**) and a detailed view of craters in a 5.8 km × 7.3 km area near the landing point.

**Figure 8:**
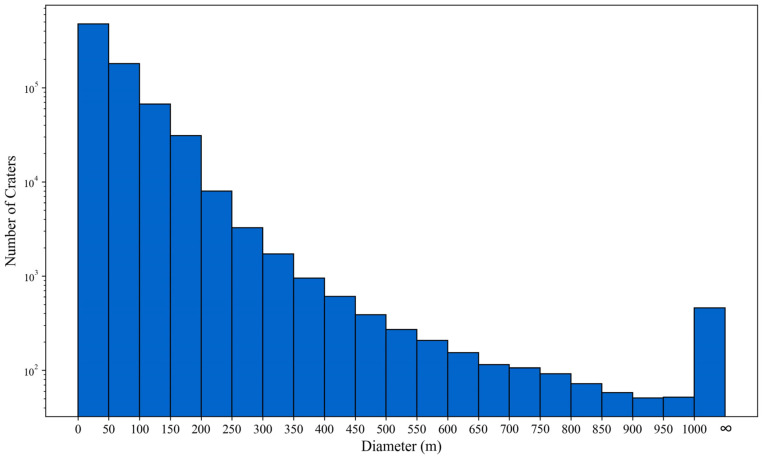
Size distribution of the impact crater diameters in the CE-6 landing area.

The final impact crater catalogue is displayed in Figure 7, and a total of 770,671 impact craters were identified within this 246 km × 135 km area. Of these, 80,825 craters are larger than 120 m. Given the large size of the database, Figure 7 only showcases craters with diameters greater than 120 m across the landing area. Meanwhile, this figure selects four evenly distributed regions (A, B, C, and D) to provide a clearer demonstration of the algorithm’s effectiveness in extracting impact craters across different terrains. Furthermore, Figure 7 enlarges a 5.8 km × 7.3 km region near the landing point to display all craters detected by the YOLOv8-LCNET algorithm. The diameters of the craters in this database range from 13 m to 19,882 m, with their distribution by diameter shown in Figure 8. In this figure, craters with diameter smaller than 50 m accounted for over 50% of the total, and the number of craters decreased exponentially as the diameter increased. These data have undergone meticulous processing and correction, ensuring high accuracy and reliability in impact crater measurements.

**Figure 9:**
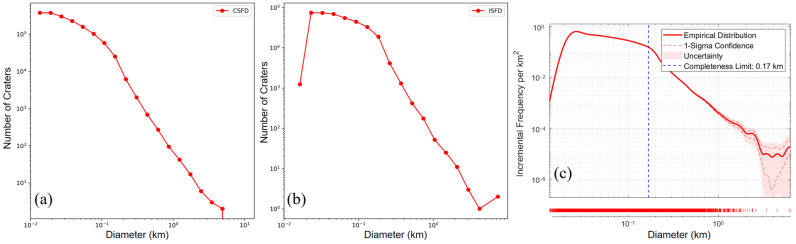
The size–frequency distributions of the impact craters in the mare region with the internal diameter of $\sqrt{2}$D in a log–log plot. (**a**) The cumulative size–frequency distribution (CSFD) of craters [51]. (**b**) The incremental size–frequency distributions (ISFD) of craters [52]. (**c**) The ISFD established by robust kernel density estimation [53].

**Figure 10:**
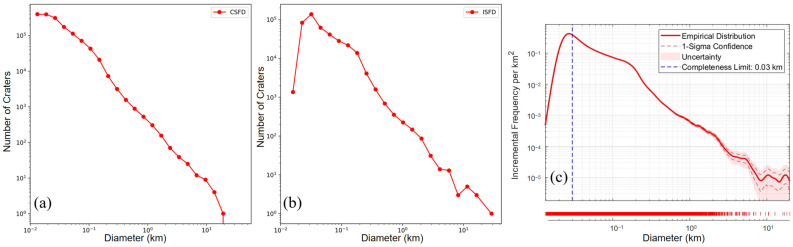
The size–frequency distributions of the impact craters in the highland region with the internal diameter of $\sqrt{2}$D in a log–log plot. (**a**) The CSFD of craters [51]. (**b**) The ISFD of craters [52]. (**c**) The ISFD established by robust kernel density estimation [53].

Based on geological differences, the CE-6 landing area is divided into two regions: the mare and the highland, as shown in Figure 1. Three different methods were applied to analyze the completeness diameters of impact craters in these two regions [51,52,53]. The size–frequency distributions of the impact craters in the established catalogue are shown in both incremental and cumulative formats in Figure 9 and Figure 10. Figure 9a–c reveal that the completeness diameters in the mare region are 20 m, 30 m, and 170 m, respectively, while Figure 10a–c show that the completeness diameters in the highland region are 20 m, 40 m, and 30 m, respectively. It can be concluded that the completeness diameter of our entire crater database is approximately 30 m, further validating the reliability of the database.

To support the safety evaluation of the CE-6 mission, we also conducted a spatial density analysis of the impact craters in the landing area, with the detailed results available in the study by Wang et al. [15]. These results guided a thorough safety assessment of the landing region. In the selection of the landing point, large craters were avoided to reduce the risk posed by their walls, which may threaten a safe landing and obstruct communication and sunlight. Therefore, the study of the impact crater spatial distribution provided a basis for the selection of the landing point for CE-6. The final selection of the CE-6 landing point was in an area with relatively low crater density.

Furthermore, after excluding secondary craters, our impact crater database was employed as a dating tool to determine the absolute model ages of different geological units within the CE-6 landing area [15]. By integrating these crater-based dating results with radiometric age data from CE-6 collected samples, the current lunar chronology models can be further refined and improved.

Since impact craters with diameters larger than 200 m are crucial for lunar chronology, our previous work published a catalogue of the impact craters with diameters larger than 200 m in this region. In this study, we further extended our work by releasing data for the impact craters with diameters greater than 30 m, which is more valuable for the geologic studies of small craters.

## 5. Conclusions

In this paper, we proposed an efficient impact crater detection model YOLOv8-LCNET. By introducing the PSA attention mechanism and GD mechanism, the model achieved a recall rate of 84.3% and a precision rate of 87.7%, and was able to identify impact craters with diameters as small as four pixels. Compared to the original YOLOv8 model, YOLOv8-LCNET significantly enhanced the ability to detect impact craters across multiple scales, offering improved accuracy in both impact crater positions and sizes. Based on this model, a database of impact craters with a completeness diameter of approximately 30 m in the CE-6 landing area has been established, containing 770,671 impact craters with diameters covering a range from 13 m to 19,882 m. The database was conducted and analyzed not only to provide engineering support for accurate landing point determination of the CE-6 lander, but also to assist in the study of lunar geology in this region.

This work primarily focuses on the CE-6 landing area, and there are plans to extend this approach to other regions. Future research will further expand the generalization ability of the model by incorporating more impact crater samples from various regions of the Moon.

## Figures and Tables

**Table 1:** Comparison of model performance before and after data augmentation.

	P	R	AP50	AP50−95	$AP_{S}$	$AP_{M}$	$AP_{L}$
Before data augmentation	0.630	0.516	0.547	0.325	0.301	0.311	0.283
After data augmentation	**0.858**	**0.828**	**0.906**	**0.675**	**0.579**	**0.675**	**0.738**

**Table 2:** Ablation experiment results of different modules.

	+PSA	+GD	P	R	AP50	AP50−95	$AP_{S}$	$AP_{M}$	$AP_{L}$	DR	GFLOPs	FPS
YOLOv8			0.858	0.828	0.906	0.675	0.579	0.675	0.738	0.199	**8.10**	**462.45**
YOLOv8	√		0.861	0.833	0.909	0.677	0.635	0.747	0.786	**0.225**	8.30	455.06
YOLOv8		√	0.876	0.841	0.919	0.710	**0.665**	0.790	0.834	0.188	17.6	431.35
YOLOv8	√	√	**0.877**	**0.843**	**0.920**	**0.712**	0.664	**0.792**	**0.836**	0.203	17.7	426.40

**Table 3:** Comparison of model performance between YOLOv8 and YOLOv8-LCNET.

	P	R	AP50	AP50−95	$AP_{S}$	$AP_{M}$	$AP_{L}$	dLo/R	dLa/R	dR/R
YOLOv8	0.630	0.516	0.547	0.325	0.301	0.311	0.283	0.07531	0.07102	0.06237
YOLOv8-LCNET	**0.877**	**0.843**	**0.920**	**0.712**	**0.664**	**0.792**	**0.836**	**0.06690**	**0.06395**	**0.05689**

## Data Availability

Impact craters with diameters greater than 30 m presented in the study are openly available in Zenodo at https://doi.org/10.5281/zenodo.14588208 (accessed on 2 January 2025).
